# Role of Female Sex Hormones in ADPKD Progression and a Personalized Approach to Contraception and Hormonal Therapy

**DOI:** 10.3390/jcm13051257

**Published:** 2024-02-22

**Authors:** Micaela Petrone, Martina Catania, Liliana Italia De Rosa, Rebecca S. Degliuomini, Kristiana Kola, Chiara Lupi, Matteo Brambilla Pisoni, Stefano Salvatore, Massimo Candiani, Giuseppe Vezzoli, Maria Teresa Sciarrone Alibrandi

**Affiliations:** 1 O.U. Obstetric and Gynecology, IRCCS San Raffaele Scientific Institute, 20132 Milan, Italy; petrone.micaela@hsr.it (M.P.); degliuomini.rebecca@hsr.it (R.S.D.); lupi.chiara@hsr.it (C.L.); salvatore.stefano@hsr.it (S.S.); candiani.massimo@hsr.it (M.C.); 2 O.U. Nephrology and Dialysis, IRCCS San Raffaele Scientific Institute, 20132 Milan, Italy; derosa.liliana@hsr.it (L.I.D.R.); kola.kristiana@hsr.it (K.K.); brambilla.matteo@hsr.it (M.B.P.); vezzoli.giuseppe@hsr.it (G.V.); sciarronealibrandi.mariateresa@hsr.it (M.T.S.A.); 3 Vita Salute San Raffaele University, 20132 Milan, Italy

**Keywords:** ADPKD, polycystic kidney, female sexual hormones, contraception, hormonal therapy, childbearing age, menopausal transition

## Abstract

This review navigates the intricate relationship between gender, hormonal influences, and the progression of autosomal dominant polycystic kidney disease (ADPKD), highlighting the limited literature on this crucial topic. The study explores the impact of female sex hormones on liver and renal manifestations, uncovering gender-specific differences in disease progression. Actually, hormonal therapy in women with ADPKD remains a challenging issue and is a source of concern regarding its potential impact on disease outcomes, particularly at the hepatic level. Notably, women with ADPKD exhibit a slower renal disease progression compared to men, attributed to hormonal dynamics. This review sheds light on the role of estrogen in regulating pathways of the renin–angiotensin–aldosterone system, revealing its complex interplay and implications for cardiovascular and renal health. Therapeutic considerations for fertile women with ADPKD, including contraception options, are discussed, emphasizing the necessity for personalized approaches. In the postmenopausal phase, the review evaluates the role of hormonal replacement therapy, considering its potential benefits and risks in the context of ADPKD. The review concludes by underscoring the imperative need for tailored treatment approaches for ADPKD patients, considering individual risks and benefits. The scarcity of literature underlines the call for further research to enhance our understanding of optimal hormonal therapies in the context of ADPKD, ultimately paving the way for innovative and personalized therapeutic interventions.

## 1. Preface

Gender exerts a significant influence on the occurrence and progression of many renal diseases, including ADPKD. ADPKD, impacting roughly 12 million individuals globally, affects both men and women equally. Mutations in PKD1 and PKD2 genes contribute to ADPKD, with gender playing a crucial role in disease manifestation and progression [1]. Women with ADPKD generally exhibit a slower progression to end-stage renal disease (ESRD) compared to men. The Predicting Renal Outcome in Polycystic Renal Disease (PROPKD) Scoring System identifies gender as a significant factor in disease progression [2].

While renal cyst growth and kidney enlargement are central to ADPKD, gender differences emerge in renal complications. Men are more prone to hypertension and extensive hematuria, while women often experience earlier and more severe polycystic liver disease, likely influenced by estrogen activity. Pregnancy, oral contraceptives, and menopausal hormone therapy can impact the disease course.

This study aims to comprehensively review the role of female sex hormones in ADPKD progression, emphasizing the need for tailored therapeutic approaches for affected women. In clinical practice, navigating challenges related to contraception and menopausal therapy for women with ADPKD is a common aspect. Hormonal therapy in women affected by ADPKD remains a complex and often debilitating aspect, the genuine risks of which have yet to be fully elucidated, requiring a nuanced understanding of the disease’s complexities.

## 2. Background

### 2.1. ADPKD

Autosomal dominant polycystic kidney disease (ADPKD) stands as the most prevalent genetic cystic kidney disorder, with 85% of cases attributed to mutations in the PKD1 gene on chromosome 16 and 10% to mutations in the PKD2 gene on chromosome 4. ADPKD diagnosis relies on familial history and ultrasound assessments, with approximately 25% of cases lacking a familial link, suggesting latent forms or novel genetic mutations [3]. Renal manifestations include bilateral cysts leading to increased renal volume and progressive renal failure, affecting around 50% of individuals by age 60. Notably, a meta-analysis indicated a less aggressive progression of ADPKD in women compared to men [4]

Beyond renal complications, ADPKD can present with high blood pressure, hematuria, mitral valve prolapses, pericardial effusion, diverticulosis, pancreatic cysts, and cerebral aneurysms. The prevalence of cerebral aneurysms is notably elevated, especially in those with a family history of aneurysms or subarachnoid hemorrhages [5,6]. The primary extrarenal manifestation involves multiple liver cysts, affecting 10% of ADPKD patients with severe polycystic liver disease (PLD). Autosomal dominant polycystic liver disease (ADPLD) and rare renal parenchymal cysts are associated with different mutations. Although hepatic involvement occurs equally in males and females, it manifests earlier and more severely in females [7].

**Figure 1:**
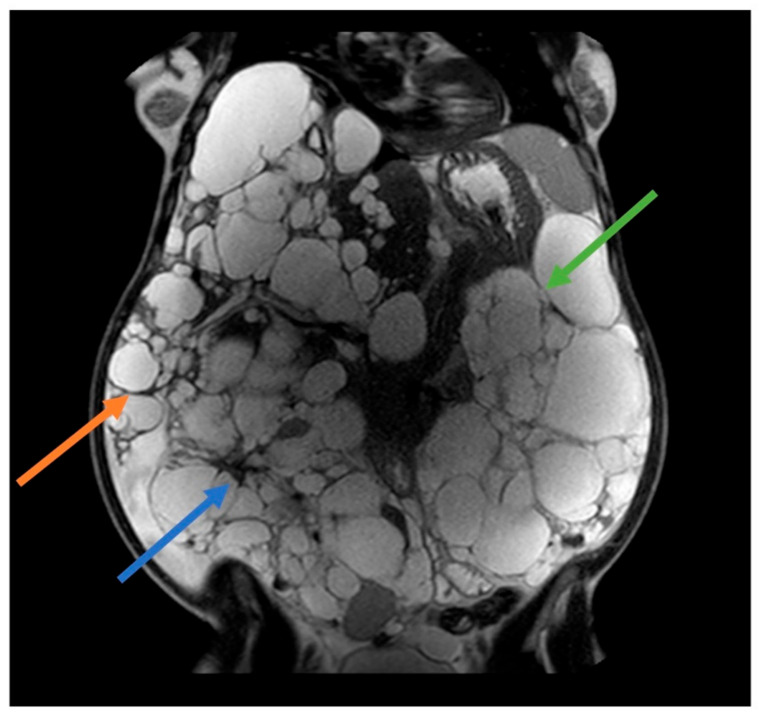
41 years old ADPKD women with nephromegaly and hepatomegaly. Two previous pregnancies and 5 years of estroprogestinic pill. Mayo Class 1 E. Orange arrow: lower margin of the right hepatic lobe; blue arrow: right kidney; green arrow: left kidney.

Most women develop liver cysts by age 60, particularly those with a history of pregnancies and/or estrogen-progestin therapy for contraception. A prospective study in postmenopausal women with ADPKD revealed the crucial role of estrogen in hepatic cystogenesis and increased liver volume, establishing it as a primary contributing factor [8]. Hormonal changes during pregnancy, specifically increased levels of estrogen and progesterone, may contribute to liver cyst enlargement and kidney cyst growth to a lesser extent. Additionally, increased renal blood flow during pregnancy could exacerbate cyst growth (Figure 1). It is worth noting that the effect of pregnancy on ADPKD can differ among individuals. Certain women may observe a marked increase in cyst size, whereas others may exhibit no notable alteration [9]. 

A crucial aspect is the involvement of a multidisciplinary team, including a nephrologist, gynecologist, and geneticist. Their aim is to offer tailored advice based on the patient’s clinical characteristics to effectively address this pathway.

### 2.2. ADPKD, Female Sexual Hormones, and Raas

#### 2.2.1. Female Sex Hormones

In recent decades, there has been a notable shift in medical research towards prioritizing women’s well-being. Previously underestimated conditions, often labeled as “hidden diseases”, have received increased attention and scrutiny. Simultaneously, there has been a heightened focus on understanding and addressing aspects of female sexual health and the physiological but impactful stages of a woman’s life, including menopause.

It is crucial to acknowledge that women of fertile age are increasingly seeking hormonal therapies, not solely for contraceptive purposes but also for managing various conditions such as abnormal uterine bleeding, endometriosis, adenomyosis, chronic pelvic pain, dysmenorrhea, polycystic ovary syndrome (PCOS), and premenstrual syndrome. Gynecologists often recommend hormonal-based treatments, although non-hormonal alternatives are also available.

The use of hormonal contraception in women with autosomal dominant polycystic kidney disease (ADPKD) has been a subject of significant debate. During the post-menopausal period, gynecological care is directed towards addressing climacteric symptoms like hot flushes and genito-urinary syndrome, along with osteoporosis prevention.

The established role of estrogen in modulating various pathways of the renin–angiotensin–aldosterone system, influencing blood pressure, and, above all, the proliferative impact of estrogen on hepatic cysts has been well documented. Consequently, ADPKD has conventionally been considered a contraindication for hormonal treatments.

Steroid Hormones and Reproductive Regulation:

Steroid hormones, including estrogen and progesterone, play a pivotal role in regulating mammalian reproduction, especially in uterine development and function. Operating primarily through gene transcription control within the uterus, these hormones exert their effects via specific receptors, acting as nuclear transcription factors. Their regulatory activity is triggered by binding steroid molecules, initiating a cascade of events that influence gene transcription. Estradiol, estrone, and estrone sulfate, varying in proportions based on menopausal status, are the primary estrogens in women’s bloodstream, with crucial roles in cellular processes, including the regulation of cell proliferation [10]. Endogenous progesterone undergoes metabolic transformation into three biologically active metabolites. Approximately 50% is converted to 5α-dihydroprogesterone in the corpus luteum, 35% undergoes hepatic metabolism to 3β-dihydroprogesterone, and 10% transforms into 20α-dihydroprogesterone [11]. Estrogen receptors (ERs), specifically ER-alpha and ER-beta in hepatocytes, have direct and indirect effects on cell proliferation. The binding of estrogen to these receptors can directly influence gene transcription related to cell proliferation, promoting cell progression through the G1 phase. Indirectly, estrogen stimulates the transcription and release of hepatocyte growth factor (HGF) and insulin-like growth factor (IGF), enhancing cell growth. Estrogen also interacts with other cell proliferation signaling pathways, including the mitogen-activated protein kinase (MAPK) pathway, potentially amplifying proliferative effects [12]. 

On the contrary, there are no data in the literature demonstrating the primary role of progesterone in the growth of liver cysts.

#### 2.2.2. Estrogen and Renin–Angiotensin–Aldosterone System (RAAS): Unraveling the Complex Interplay

The renin–angiotensin–aldosterone system (RAAS) intricately regulates cardiovascular and renal functions, exerting a profound impact on arterial blood pressure (BP) regulation. Elevated RAAS activity is implicated in various cardiovascular and renal diseases, including hypertension. Estrogen is a key suppressor of RAAS, and its absence, as seen in menopause, may contribute to heightened RAAS activity [13,14,15,16]. RAAS modulation exhibits variations across the menstrual cycle. While the specific role of the RAAS in the follicular and ovulation phases of the menstrual cycle may not be extensively studied, in the luteal phase, characterized by high estrogen and progesterone levels, RAAS activity increases. However, during simulated orthostatic stress, estrogen-induced decreases in tissue responsiveness to RAAS or opposing vasodilatory effects may prevent the maintenance of mean arterial blood pressure [17,18]. Postmenopausal women (PMW) show RAAS activation during orthostatic stress, and estrogen therapy restores RAAS responsiveness [19]. Interestingly, despite RAAS activation, systolic blood pressure remains lower with estrogen therapy. Estrogen’s complex effects include upregulating angiotensinogen gene expression, altering renin concentrations, and increasing vasodilators like cardiac atrial natriuretic peptide (ANP).

Estrogen generally elevates angiotensinogen levels while decreasing renin, ACE activity, AT(1) receptor density, and aldosterone production. It also activates RAAS counterparts like natriuretic peptides, AT(2) receptor density, and angiotensinogen. Progesterone competes with aldosterone for mineralocorticoid receptors, while testosterone, which is less understood, appears to increase renin levels and ACE activity [20].

**Figure 2:**
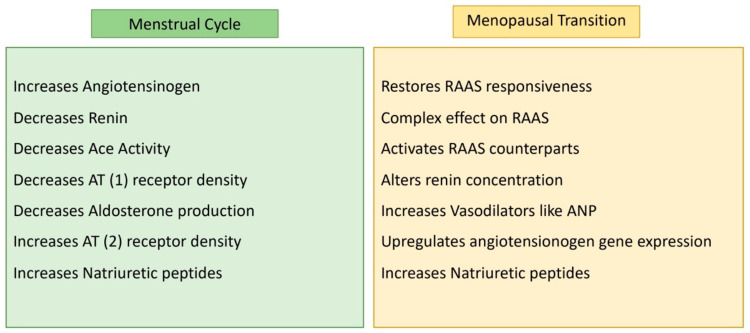
RAAS activity according to female hormonal changes.

These hormonal effects on RAAS contribute to gender differences in cardiovascular and kidney diseases. Understanding this complex interplay sheds light on conditions influenced by RAAS dysregulation (Figure 2).

### 2.3. The Role of Estrogens on Renal Function

Contrary to expectations, estrogen emerges as a safeguard against renal failure progression in females with autosomal dominant polycystic kidney disease (ADPKD). Although this insight originates from a mouse model, it reveals critical mechanisms [17]. In ADPKD, males face an elevated risk of progressing to end-stage renal disease compared to their female counterparts. 

It is also worth noting that males are more prone to kidney stones than females and that kidney stones may accelerate disease progression. Estrogen is a key player in the expression of osteopontin, a defense against kidney stones [21].

In a groundbreaking finding, male sex hormones are implicated in stimulating renin–angiotensin–aldosterone system (RAAS) activation and endothelin-1 (ET1) release. In contrast, estrogen intervenes by suppressing this axis and instigating the upregulation of vascular endothelial growth factor (VEGF), thereby preserving renal function [22].

Recent evidence underscores the pivotal roles of chloride channels, specifically protein kinase A and transmembrane calcium channel 16A (TMEM16A), in ADPKD pathology. The TMEM16A promoter region houses androgen-responsive elements crucial for testosterone-dependent regulation. This mechanism holds promise for mitigating renal cyst growth in women [23].

While TMEM16A expression displays variations, cystic fibrosis transmembrane conductance regulator (CFTR) expression appears diminished in ADPKD women. This decrease in CFTR expression is attributed to estrogen-dependent regulation. Understanding these subtleties is essential, given the implications for cyst development [24]. Recent studies point to the possibility that TMEM16A expression and hormonal regulation contribute to a more severe phenotype in men with ADPKD. The observed heightened cell proliferation, driven by increased intracellular calcium levels, likely underlies a more severe cyst-related phenotype, emphasizing gender-specific differences in renal calcium homeostasis [23].

### 2.4. Early Menopause as a Risk Indicator

Compelling evidence indicates that women experiencing early menopause (before age 45) face an elevated risk of developing renal failure. This underscores the intricate interplay between hormonal regulation and ADPKD severity. On the flip side, the impact of estrogens on liver cyst involvement and growth, as mentioned, seems to be detrimental.

Considering what has been discussed so far and in light of the scientific evidence and literature, we can attempt to outline a therapeutic approach aimed at maximizing benefits while minimizing potential risks.

## 3. Possible Therapeutic Strategies for ADPKD Patients

### 3.1. Childbearing Age

#### 3.1.1. Copper Intra-Uterine Device 

When ADPKD patients’ needs are limited to contraception, we consider the copper intra-uterine device (IUD) the best choice. The copper IUD is considered to be the gold standard for ADPKD patients, being the only non-hormonal option currently available. It is a long-acting contraceptive device and was approved by the FDA in 1988. It consists of a polyethylene T-shaped device wrapped in copper wire [25]. The precise mechanisms of action of non-medicated IUDs are still sometimes not completely known. However, they create an unfavorable environment for pregnancy onset. First of all, the release of copper ions inhibits sperm mobility. Secondarily, the presence of a foreign device causes an inflammatory response, which has a spermicidal effect. It is important to underline how ovulation is not inhibited by the presence of copper IUDs [25,26,27,28]. Therefore, this treatment is not effective for some important pathologies in women, such as endometriosis, dysmenorrhea, menorrhagia, or PCOs; the latter in fact are very common reasons for women to use hormonal-based treatments [29]. 

Copper-IUD use is well tolerated by patients; however, side effects are common; some of them improve over time, while others do not. They mainly include spotting, dyspareunia, dysmenorrhea, cramping, backache, vaginitis, prolonged periods, and, rarely, spontaneous expulsion. Rarely, side effects cause early removal of the device. Complications related to IUD insertion include an increased risk of pelvic inflammatory disease, uterine perforation, mispositioning, and pain and/or bleeding post-insertion. Another aspect to be taken into consideration is the possible pain of IUD insertion in nulliparous women and young patients. IUD insertion is contraindicated whenever a woman has anatomical anomalies affecting the uterus (e.g., bicornuate uterus, fibroids altering the endometrial cavity, etc.) [27].

Copper IUDs still represent an effective alternative in cases of contraindicated hormonal treatment, such as hypertension, obesity, breast cancer, and deep venous thrombosis. Most importantly for this review, copper IUDs can be used in patients with hypertension and both benign and malignant liver tumors, such as focal nodular hyperplasia, hepatocellular adenoma, and hepatoma [30]. 

For this reason, the use of copper IUD as a contraceptive method for ADPKD is safe, easily reversible, inexpensive, highly effective, and long-acting.

#### 3.1.2. Levonorgestrel-IUDs

Levonorgestrel-IUDs can be an adequate treatment option for endometriosis, adenomyosis, chronic pain, irregular periods, or abnormal uterine bleeding in ADPKD patients. 

There are three different types of progestin-impregnated intrauterine systems.

- The 52-mg Levonorgestrel-IUD (LNG-IUD) retains Levonorgestrel (52 mg) and releases 20 mcg of LNG per day. The two types available in the US are Mirena and Liletta, and they can be used for a maximum of 8 years (FDA-approved).
- Another type of LNG-IUD contains 19.5 mg of LNG (Kyleena) and releases 13 mcg per day. It can be used for up to 8 years.
- The third type is the 13.5 mg LNG-IUD (Skyla), also called “low-dose LNG-IUD”, which releases 8 mcg of LNG per day and can be used for only 3 consecutive years.

These devices act mostly by thickening the cervical mucus and changing the pattern of the endometrium [28,29,30,31]. Sometimes they can also suppress ovulation, but most women continue to ovulate with LNG-IUD, especially the low-dose one [28]. Patients often become amenorrheic while the LNG-IUD is in place. LNG-IUDs are effective as contraceptives, but they are also employed for the management of other medical conditions. For example, 52 mg of LNG-IUD represents the current standard non-surgical treatment of endometrial pathologies such as endometrial hyperplasia, endometrial intraepithelial neoplasia, and grade 1 endometrioid endometrial cancer [30,31]. Because of its endometrial suppressing action, LNG-IUD is considered a treatment for menstrual disorders, above all menorrhagia and dysmenorrhea. More specifically, LNG-IUDs are proven to be effective in reducing heavy menstrual bleeding (HMB), more so than oral contraceptives [31,32,33,34,35]. LNG-IUDs also play an important role in decreasing uterine volume in patients affected by adenomyosis and uterine fibroids [34]. LNG-IUD has shown benefits in treating endometriosis-related pain, as per the most recent ESHRE endometriosis guidelines [31,33,35,36]. The most reported side effects of LNG-IUD are caused by the progestin and include headaches, nausea, breast tenderness, and decreased libido. 

#### 3.1.3. Combined Estrogen-Progestin Oral Contraceptives (COCs) 

COCs suppress ovulation by inhibiting the gonadotropin-releasing hormone (GnRH) from the hypothalamus, inhibiting follicle-stimulating hormone (FSH) and luteinizing hormone (LH), and disrupting the LH surge in the middle of the cycle. They also cause endometrial atrophy, an increase in cervical mucus thickness, and an impairment of tubal mobility. All these mechanisms of action together contribute to the contraceptive effect [37]. There is an enormous variety of COCs on the market nowadays, differing in the type and dosage of progesterone and estrogen. COCs are recognized to have numerous non-contraceptive benefits, such as: pelvic pain relief in patients affected by endometriosis; treatment of PCO-related signs (acne and hirsutism); reduction of dysmenorrhea, menorrhagia, and consequently iron deficiency anemia; reduction of the risk of ovarian, colorectal, and endometrial cancer; reduction in the risk of benign breast disease; and reduction of ovary cysts [38,39,40,41,42]. However, hepatic diseases are a contraindication for the use of COCs, and, therefore, they cannot be administered in patients affected by ADPKD with hepatic involvement [30]. In an interesting recent study focused on the role of estrogen-containing oral contraceptives in the severity of liver disease in women affected by polycystic liver disease, premenopausal women demonstrated increased hepatic cyst volume associated with COC use, specifically a 15.5% increase in hepatic volume in 10 years of use. Concluding, it appears a safe choice for PLD patients not to intake exogenous estrogens [43].

#### 3.1.4. Vaginal Contraceptive Ring

If hormonal treatment inducing ovulation inhibition is strictly necessary, extremely well-pondered and informed options can be considered.

The vaginal contraceptive ring is a combined hormonal option. The mechanism of action is the same as that of the estroprogestinic pill: mainly, the suppression of ovulation. Vaginal rings are plastic polymer rings releasing ethinyl estradiol and the third-generation progestinic (etonogestrel). Hormonal absorption occurs directly through the vaginal mucosa into systemic blood circulation. Vaginal rings are kept in place for 21 days and removed for 7 days. During the discontinuation week, endometrial bleeding occurs. They present the same side effects of combined oral contraceptives (COCs): headache, breast tenderness, nausea, mood changes, and vaginal discharge [30,44,45]. They also present the same contraindications: high BMI, migraine, history of deep venous thrombosis, hypertension, and breast cancer [30]. Interestingly, for this review, vaginal rings present a key characteristic: their local absorption. This implies a diminished risk of systemic estrogen-related adverse effects. Their local absorption allows them to bypass the gastrointestinal and liver passages, and, moreover, they have been observed to maintain a stable estrogen level during the day. In contrast, indeed, classic combined oral contraceptives (COCs) present hormonal concentration oscillations in the blood during the day based on their time of assumption [37,45]. This important characteristic of the vaginal ring makes this treatment the only hormonal treatment that may eventually be considered for ADPKD women. Their lower hormonal systemic dosage, delivered at stable levels, bypassing the hepatic metabolism, may represent a chance for personalized, tailored treatment in selected cases with strict follow-up [46].

#### 3.1.5. Progestogen-Only Contraceptives (POPs)

Progestogen-only contraceptives are considered a safer alternative to traditional methods involving external estrogens, specifically ethinylestradiol. POPs are widely utilized due to their noninvasive and easily reversible nature as a contraceptive method. POPs function by inhibiting ovulation and modifying cervical mucus. Desogestrel 75 mg, given continuously for 28 days, achieved optimum results as follows: a 99% ovulation inhibition rate. POPs are considered the primary choice for hormonal contraception in targeted groups of patients, such as older women, breastfeeding patients, and individuals for whom estrogen-based COC pills are not suitable [47,48]. Unlike COCs, POPs can be used safely by women with diagnosed thrombophilia. However, there remain contraindications for the use of POPs, which include patients with venous thromboembolic disease, non-investigated vaginal bleeding, severe liver disease, and individuals with sex-steroid-sensitive cancers. Studies have shown that the rates of ovulation inhibition are similar between COCs and desogestrel-containing POPs [WHO]. It should be noted that some authors consider progesterone-only pills a gold standard treatment against endometriosis [49]. In PCOs, instead of COCs representing the gold standard, particularly for women desiring contraception with hyperandrogenism-related symptoms, POPs are only recommended for those with contraindications to COCs [50].

Considering the safety profile of POPs, specifically regarding the thrombotic risk and the limited action of progesterone on the renin-angiotensin-aldosterone system, ADPKD patients without hepatic involvement may be suitable for their use. It is evident that a risk-benefit balance, together with an informed consensus and strict follow-up, is mandatory.

### 3.2. Menopausal Transition

With the general population living longer, research regarding menopause is increasing. More and more women seek treatment for the discomfort caused by this condition. Most women complain of extremely bothersome, sometimes invalidating, climacteric symptoms. Hormonal replacement therapy (HRT) is considered the gold standard for the treatment of menopause-related symptoms. Multiple studies over the years and decades have demonstrated the fundamental importance of this therapy for what concerns quality of life; furthermore, some HRTs reduce the risk of life-threatening conditions such as colorectal cancer [51,52]. HRT has the best risk–benefit balance when administered to symptomatic women younger than 60 years of age or in the 10 years after menopause. HRT has optimal results in managing all symptoms and also reduces the risk of osteoporosis. The North American Menopause Society considers HRT to be the gold standard treatment for osteoporosis in climacteric symptomatic women within this window of opportunity [53]. Moreover, the presence of kidney disease appears in multiple studies to be an indirect cause of early menopause. Pathophysiology is poorly understood. It is clear that low estrogen levels after menopause affect renovascular physiology; however, due to the lack of large prospective studies, the precise pathophysiology is still unclear. Kidney transplants in early menopausal women may even bring menses back, as a clear demonstration of the correlation. However, what is most important is to underline the cardiovascular risk present in post-menopausal women (especially in cases of early menopause), which is clearly worsened by the presence of kidney disease [54,55]. Furthermore, growing evidence is revealing how vasomotor symptoms may be biomarkers of cardiovascular disease risk, inducing physicians to pay more attention to this group of patients regarding whether to prescribe HRT [56,57]. The safety profiles of all different HRT therapies have improved over time; however, all treatments include the use of estrogen in diverse formulations and dosages. The indications and contraindications of HRT are now well defined by the major menopause societies. Currently, all symptoms of menopause can be addressed by treatments tailored to each symptom [51,58,59,60]. Vasomotor symptoms (VMS) can be partially controlled by natural preparations, although they are rarely effective, and patient compliance is consequently limited. Further evidence demonstrates the efficacy of regular physical activity in reducing hot flushes [61,62]. Vulvo-vaginal atrophy can be treated by hyaluronic acid-based ointments and lubricants. Vitamin D supplementation is vitally important for osteoporosis prevention [63]. VMS remains the most bothersome condition impacting menopausal women’s quality of life; however, a new, specific treatment is imminent. In 2023, the FDA approved the use of a new drug called Fezolinet specifically for vasomotor symptoms. Fezolinet is a neurokinin-3 receptor (NK3R) antagonist. NK3R plays a fundamental role in modulating the thermoregulatory center, triggering the so-called vasomotor response. This new drug stops the development of this response. This is a totally non-hormonal alternative. Given the presence of this new drug on the market, in the author’s opinion, clinicians should consider avoiding HRT in ADPKD women due to the unfavorable risk-benefit ratio. With new treatments available, ADPKD in post-menopausal patients should lead to personalized, efficacious therapies tailored to each symptom [64].

Given the complexity of ADPKD and the potential risks associated with hormone therapy, non-hormonal interventions play a key role in the possible management of menopausal symptoms. 

Lifestyle changes, such as dietary changes and regular exercise, can contribute to overall well-being. In addition, cognitive-behavioral therapies may help women manage mood disorders.

Postmenopausal women have an elevated risk for osteoporosis, and this risk is exacerbated in women with ADPKD due to renal insufficiency associated with changes in calcium and phosphorus metabolism. Adequate calcium and vitamin D supplementation, along with regular bone density monitoring, are essential components of a comprehensive management plan for these women. However, there is currently a consistent lack of data regarding the impact of hormonal therapy on ADPKD patients, as these medications are not used in clinical practice. The aim of this review is to pave the way to establishing a safety profile for treatments for ADPKD patients whenever possible.

## 4. Conclusions

**Figure 3:**
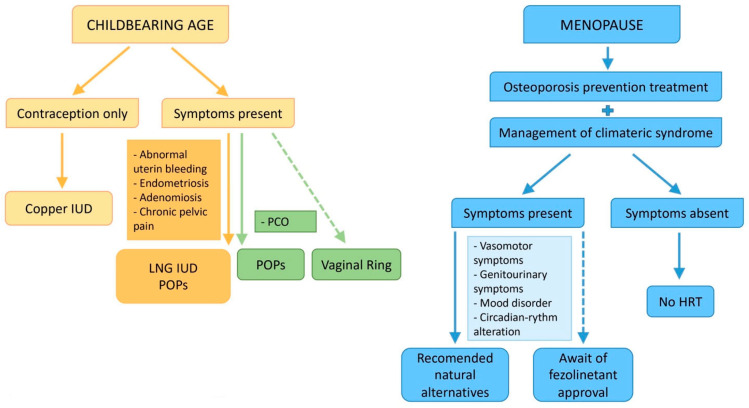
Possible therapeutic algorithm.

Navigating hormone therapy and contraception in ADPKD requires personalized care (Figure 3). Copper IUDs are favored for contraception, while hormone therapy needs cautious assessment. Progestin-only options are recommended for endometriosis and PCOS. Non-hormonal alternatives like Fezolinet are promising for menopause. Personalized assessment is key, with ongoing research aimed at refining therapy in ADPKD. Together, we aim for improved patient care, ensuring each journey is guided with care and precision.
